# Time-driven effects on processing grammatical agreement

**DOI:** 10.3389/fpsyg.2013.01004

**Published:** 2013-12-30

**Authors:** Mikael Roll, Sabine Gosselke, Magnus Lindgren, Merle Horne

**Affiliations:** ^1^Department of Linguistics and Phonetics, Lund UniversityLund, Sweden; ^2^Department of Psychology, Lund UniversityLund, Sweden

**Keywords:** agreement, grammatical dependency, ERP, left anterior negativity, LAN, short-term memory, working memory, P600

## Abstract

“Agreement” is a grammatical relation between words; e.g., the verbal suffix –*s* reflects agreement with a singular subject (*He run-s*). Previous studies with time intervals under 2.5 s between disagreeing words have found a left-lateralized negative brain potential, arguably reflecting detection of the morphosyntactic violation. We tested the neurophysiological effects of number agreement between the first and last word in sentences at temporal distances between 1.75 and 3.25 s. Distances were varied by visually presenting sentences word by word at different rates. For distances under 2.5 s, a left-lateralized negativity was observed. At a 3.25-s interval, an anterior, slightly right-lateralized negativity was found. At an intermediate distance of 2.75 s, the difference between disagreement and agreement at left electrodes correlated with participants' working memory span. Results indicate that different brain processes occur when agreement involves agreement domains approaching and exceeding 3 s than when the agreement dependency involves shorter temporal intervals.

## Introduction

### A time window for formal processing

Ever since the studies by Sachs (1967, 1974), it has been known that grammatical information decays in short-term memory during the first few seconds after language exposure. To test whether different kinds of linguistic information decayed at different rates in short-term memory, Sachs (1974) let participants read texts containing sentences like *A wealthy manufacturer, Mathew Bolton, sought out the young inventor*. At different time intervals after reading the original sentence, participants read a test sentence that was either identical or contained a slight change with regard to the original sentence, including word order (… *sought the young inventor out*), choice of words (*A rich manufacturer*…), or propositional meaning *The young inventor sought out a wealthy manufacturer, Mathew Bolton*. When test sentences were presented immediately after the original sentence, all alterations were identified above chance. However, already after the shortest delay (4 s), word order and word choice had been forgotten, whereas participants still performed above chance as regards changes in propositional content at delays of 80 s. Sachs suggested that the rich representation of sentences involving their grammatical form is recoded into a sparser semantic format during the first seconds following language exposure.

The rapid forgetting of grammatical information could be a consequence of a more general cognitive mechanism for information processing. Thus, Pöppel (1997) has proposed that integration of perceptual information is limited to a time window of ~3 s. Only semantic information extracted from this temporally restricted perceptual unit would remain after 3 s. In addition to Sachs' (1967, 1974) findings for grammar processing, results from studies on prosodic phrasing support a short time window for integration of formal aspects of language. Thus, both speakers (Vollrath et al., 1992; Horne et al., 2006) and readers (Roll et al., 2012) seem to show a preference for prosodic phrases under 3 s, possibly to be able to integrate all words into phrases while phonological and morphological information is still activated. Similarly, word list recall and mismatch negativity (MMN) studies have shown decay of phonological memory traces starting within 2–3 s after reading or hearing words and sounds (Peterson and Peterson, 1959; Baddeley, 1966; Baddeley et al., 1975; Mäntysalo and Näätänen, 1987; Sams et al., 1993). If, however, integration of phonological and grammatical information is limited to a short time window, it is unclear how grammatical dependencies are processed if the dependent forms are separated by more than ~3 s. It could perhaps be the case that higher-level semantic information and pragmatic processing strategies are engaged when formal grammatical information has faded away after ~3 s. The present study investigated how number agreement is affected by different word presentation rates, producing different temporal distances between agreeing words.

### Number agreement

**Figure 1:**
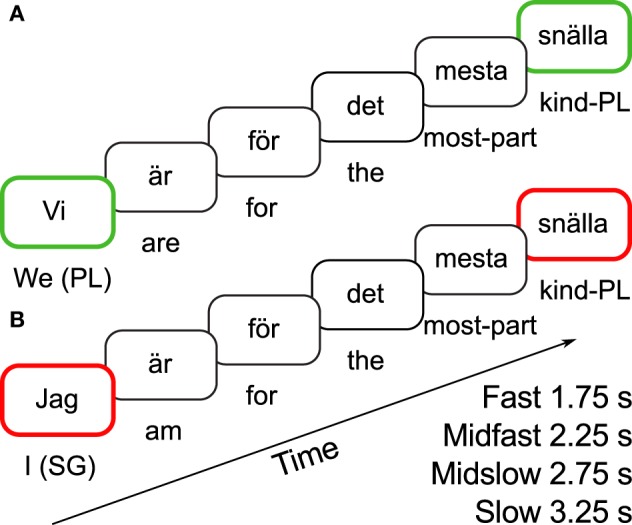
**Example sentences showing (A) agreement and **(B)** disagreement between grammatical subject and sentence-final predicate adjective, as well as the time intervals between subject pronoun and predicate adjective resulting from the different rates of word presentation**.

Grammatical agreement is a language phenomenon where a word is explicitly marked for its grammatical relationship with another word in a sentence (Wechsler, 2009). For instance, the singular -*s* suffix in *She know-s* as opposed to *They know* shows the relation between the singular grammatical subject *she* and the verb *knows*. In Swedish, there is no subject-verb agreement. However, in contrast to English, adjectives agree in number with the noun phrase (NP) they modify. Thus, used together with a singular NP like *flicka* “girl” in *snäll* *flicka* “kind girl,” the adjective *snäll* “kind” does not have any suffix. However, when modifying a plural NP like *flickor* “girls” in *snäll-a flickor* “kind-pl girls,” the adjective receives an-*a* suffix, indicating that it is modifying a plural NP. Figure 1 illustrates the kind of test sentences used in the present study to investigate effects of agreement/disagreement in different time-domains. This was made possible by interspersing text between the subject pronoun and predicate adjective without interfering with the agreement relation.

When reading the subject *vi* “we” in Figure 1, its grammatical form as well as possible referents are likely to be activated in the reader's short-term memory. When encountering the adjective *snäll-a*, the suffix-*a* can be thought to cue the reader to search in short-term memory for a grammatical form matching its plural specification. In this way, the modified pronoun *vi* can easily be detected based on its grammatical form. However, around 3 s after reading *vi*, its grammatical form might have vanished in short-term memory, and only the semantic or propositional content associated with “we are… ” might be available. Therefore, when the agreement morpheme on the adjective cues search, no suitable grammatical form would be found in short-term memory. In this case the reader might be expected to extend search for a suitable item outside the 3-s time window based on the propositional content of the sentence available at that point (“we are…”).

### ERP effects of number agreement

#### LAN

Difference in processing agreement in terms of grammatical or semantic information at different time intervals could be reflected in different brain responses to agreement violations. Reading words disagreeing in number or gender has generally yielded a left anterior negativity (LAN) between 300 and 450 ms followed by a late posterior positivity (“P600”). Left-lateralization of the negativity was clear in studies involving distances between onsets of disagreeing words under 2.5 s due to the presentation rate and number of words in the sentences (Osterhout and Mobley, 1995; Coulson et al., 1998; Gunter et al., 2000; Angrilli et al., 2002; Barber and Carreiras, 2005; Roehm et al., 2005; Martín-Loeches et al., 2006; Silva-Pereyra and Carreiras, 2007; Molinaro et al., 2008; Mancini et al., 2011; O'Rourke and Van Petten, 2011). The LAN has been interpreted as indexing the detection of a morphosyntactic violation. This is what would happen if an agreement morpheme is encountered and only a mismatching grammatical form is found in short-term memory. The later positivity would rather reflect an attempt to repair the broken grammatical relation (Friederici, 2002). However, if the reader finds no grammatical form of the right word class in short-term memory, other brain responses might be expected. These responses would reflect extended search for previously encountered grammatical forms based on the semantic or propositional content of the sentence.

Thus, if readers encounter the agreement suffix in *snäll-a* “kind-pl” and more than 3 s have passed since reading the mismatching pronoun NP *jag* “I” in ∗*Jag är snäll-a*^1^ “I am kind-pl,” a brain response indexing the extended search to track the grammatical form of the preceding pronoun would be expected. This brain reaction could give a different ERP response as compared to the left-lateralized negativity found for shorter intervals. In effect, a study on Hungarian using a distance of 4 s between agreeing words found a central anterior negativity peaking at around 390 ms for number agreement violations (Jolsvai et al., 2011). The lack of left-lateralization of the negativity in this study could be thought to be due to a reduced contribution of grammatical form in agreement processing at this long temporal distance. Other studies involving intervals longer than 3 s have shown similar results. For instance, Kaan (2002) obtained a broadly distributed negativity for number disagreement in English at distances varying between 2.1 and 3.7 s due to different numbers of intervening words. However, since Kaan (2002) presented different temporal distances together, it is difficult to tease out whether there were distinct effects at different time intervals. Further, Spanish person agreement violations with a constant distance of 3 s between the critical words yielded a mainly anterior, but slightly left-lateralized, negativity for disagreeing words (Hinojosa et al., 2003). A problem with interpreting the results of this study in terms of temporal length of agreement domains is, however, that an interspersed agreeing participle interfered in the agreement relation investigated. Another study on Spanish person agreement tested violations where agreeing words were separated by distances of 500 or 2500 ms, depending on whether a relative clause had been inserted or not (Martín-Loeches et al., 2005). At 500 ms, the authors obtained a LAN for agreement violation. At the 2500 ms distance, however, the negativity disappeared.

Summing up these previous results, it can be said that a LAN with a clear left-lateralization has only been found at temporal distances under 2.5 s. Studies involving agreement domains over 3 s long have found a negativity with a more central, mostly anterior distribution. In other words, there is support for the assumption of dividing line between the brain responses to grammatical agreement for different temporal domains. The transition phase seems to occur in a time period between 2 and 3 seconds between agreeing words. A possible confound for interpretation of previous results in terms of fading of grammatical form, however, is the fact that studies comparing different temporal distances involved in agreement have manipulated distance by varying the number of interpolated words. Different numbers of words is likely to have a direct effect on working memory load, which might give rise to difference in topographical distribution of ERPs per se. Finally, somewhere between 2 and 3 s, there seems to be a gray zone where agreement violation does not yield any negativity. This might be because of different individual effects canceling out each other at the group level. Thus, it could be that grammatical forms fade more slowly in readers with greater working memory span, so that agreement violation might still produce a left-lateralized negativity when intervals come closer to 3 s, whereas readers with shorter memory span might search beyond the items activated in working memory even before intervals reach 3 s.

#### LAN and MMN

Number agreement has been investigated in MMN studies as well. Pulvermüller and Shtyrov (2003) used an auditory oddball paradigm to investigate number agreement. They obtained two MMN effects for a deviant stimulus (*We comes*) consisting of an agreement violation occasionally inserted among a large number of repetitions of the corresponding correct word sequence (*We come*). The first MMN appeared between 100 and 150 ms after the incorrect suffix, and the second MMN had a peak around 350 ms. The results have been replicated in other studies carried out on other languages (Shtyrov et al., 2003), confirming the automaticity of the process (Hasting et al., 2007; Hasting and Kotz, 2008; Pulvermüller et al., 2008) as well as its specific sensitivity to grammatical rules (Pulvermüller and Assadollahi, 2007).

Pulvermüller and Shtyrov (2003) explained their results in light of a neurobiological model involving a “neuronal sequence detector.” The sequence detector is assumed to be an ensemble of neurons responding to events of two classes appearing in a specific order. For example, upon hearing a pronoun like *she* from the class of third person singular words, the sequence detector would activate the related agreeing verbal suffix –*s* in *She comes*. This would prime the second word, which is known to reduce the MMN. In *We comes*, the verb form had not been primed by a third person singular word, and therefore produced an increased MMN.

It can be thought that the LAN often found in a 300–450 ms time window for agreement violations might correspond to the second MMN peak. Pulvermüller and Shtyrov (2003) argue that a reason why an early negativity is often not found for agreement violations could be great physical variability of the stimuli. Stimulus presentation modality is another important factor. In other words, the first left anterior peak might be specific to auditory stimulus presentation, whereas the second might be modality independent. Indeed, identical visual stimuli violating grammatical word order have been seen to evoke an early posterior negativity in the occipital cortex (e.g., Roll et al., 2007). It should be kept in mind that an assumed sequence detector would have to be able to cope with non-adjacent grammatical dependencies, since, as shown in the present study, several words might be interspersed between the words involved in the agreement relation. Another aspect that should be mentioned is that, unlike verbs, adjectives are not obligatory sentence constituents. Therefore, the subject pronoun cannot be used to predict an upcoming predicative adjective. In other words, a sequence detector might also have to involve “backwards search” in order to resolve the agreement relation.

### Current study

The present study investigated whether the brain response to number disagreement would change when the interval between agreeing words approached and exceeded 3 s. For this purpose, participants read Swedish sentences involving valid pronoun-predicate adjective agreement and violation of such agreement. In Swedish, adjectives modifying plural NPs like the pronoun *vi* “we” have an-*a* suffix, as in *snäll-a* “kind-PL,” (Figure 1A). Adjectives modifying singular NPs do not have any suffix: *Jag är snäll* “I am kind.” Figure 1B thus shows disagreement between the singular pronoun *jag* “I” and the plural form of the adjective *snäll-a* “kind-PL.” A copular verb like *är* “am/are” and one of twenty adverbial phrases such as *för det mesta* “for the most part” were inserted between the pronoun and the adjective in the test sentences. The interpolated material was created in order to be able to co-occur with all pronoun-adjective pairs, and insertion of this material was random to avoid any predictability. To allow varying the time interval between agreeing words without changing the number of intervening words, we presented the same kind of sentences visually word by word at four different presentation rates: “fast,” “midfast,” “midslow,” and “slow” (350, 450, 550, and 650 ms/word, respectively). This created a distance of 1.75, 2.25, 2.75, or 3.25 s between the onsets of the (dis)agreeing words at the different respective presentation rates. The experiment design is illustrated in Table 1. A possible problem would have been if the task influenced the participants to actively remember the subject, and thus repeat the word silently during reading. Therefore, rather than using a grammaticality judgment task, the task was to judge after each trial whether a certain word had appeared in the sentence or not. Moreover, the words involved in the agreement relation were excluded from the task. To minimize lexical semantic associations that might also enhance the memory of the initial subject, only pronouns were used. All pronouns were first person^2^.

**Table 1:** **Experiment design**.

	**Presentation rate**			
	**Fast**	**Midfast**	**Midslow**	**Slow**
Agree	AgrFast	AgrMidfast	AgrMidslow	AgrSlow
Disagree	DisFast	DisMidfast	DisMidslow	DisSlow

*There were four presentation rates, and pronoun-adjective pairs complying with agreement rules (agree) or violating agreement (disagree)*.

At the two faster rates, the grammatical form of the pronoun would be expected to be present in short-term memory when the adjective is read and serve as a basis for agreement processing. Therefore, a LAN would be expected in cases of mismatch. At the slow rate, the grammatical form of the pronoun would be expected to have faded after 3.25 s, and an extended search for an agreeing word would be thought to take place. Hence, a brain response different to that at the faster rates would be expected, possibly with more right-hemisphere involvement due to pragmatic inference (St George et al., 1999). The distance between (dis)agreeing words produced by the midslow rate of word presentation (2.75 s) should be in the upper limit for the grammatical form of the subject to still be available in short-term memory. At this rate, variability between participants with different working memory capacity might be expected. Thus, participants with a greater working memory span could be thought to have greater capacity for form-based agreement processing in a long time window. If the time grammatical forms are activated depends to some degree on individual working memory capacity, there may therefore be a correlation between working memory span and LAN effect at the midslow speed of word presentation. To test this, participants' working memory was measured using the Automated Running Span task (Broadway and Engle, 2010).

## Method

### Participants

Twenty-eight right-handed, native speakers of Swedish participated. Three were excluded due to excessively noisy EEG. Mean age was 22.5 years, *SD* = 3.25, 14 were female.

### Materials and procedure

Sentences were presented word by word in white font against a black background at the center of a computer screen. Presentation rate (SOA “Stimulus Onset Asynchrony”) included a 30 ms blank screen between words, i.e., 350 ms/word involved 320 ms word presentation and 30 ms blank screen, etc. The last word had a presentation duration of 620 ms at all speeds, and was followed by a 1030 ms blank screen. Ten different adjectives were used in both singular and plural forms, giving 20 different word forms per condition, each used twice, yielding 40 trials per condition. The subject-adjective pairs were pseudorandomly distributed over 6 blocks, keeping equiprobability of conditions within blocks. The protocol was approved by the Swedish Research Council and followed the Declaration of Helsinki.

### Electroencephalography

A 129-channel HydroCel Geodesic Sensor Net from Electrical Geodesics Incorporated (EGI) recorded the EEG at 250 Hz sampling rate. Band-pass filter with cutoff frequencies 0.01–70 Hz was used online, and a 0.1–30 Hz filter was applied offline. Impedances were kept below 50 kΩ (manufacturers recommendation, high impedance amplifiers). CZ was used as online reference, and average re-referencing was computed offline. Ground reference had a centroparietal location. Forty epochs of 750 ms were extracted per condition, using a pre-stimulus time window of 100 ms for baseline correction. Epochs exceeding ± 100 μV after compensation for eye artifacts using Independent Component Analysis (Jung et al., 2000) were rejected, *M* = 6.0, *SD* = 5.2. Participants with over 40% rejected trials were excluded from analysis. For each speed, ERP averages of six channel areas in the time windows 300–450 ms, and 600–750 ms were submitted to a repeated measures ANOVA with the factors agreement (agree, disagree), antpost (anterior, posterior), and laterality (left, mid, right). Significant and marginal interactions were broken down by the topographical factor. Greenhouse-Geisser correction was used when applicable (uncorrected values are shown within parentheses). All significant effects are reported. Channel areas were Left Anterior (electrodes 26, 27, 33, 34, 35, 39, 40), Mid Anterior (5, 6, 10, 11. 12, 16, 18), Right Anterior (2, 109, 110, 115, 116, 122, 123), Left Posterior (41, 45, 46, 47, 50, 51, 58), Mid Posterior (61, 62, 67, 71, 76, 77, 78), and Right Posterior (96, 97, 98, 101, 102, 103, 108).

## Results

### Behavioral results

Accuracy was high, *M* = 98.74% correctly answered questions with no significant difference between conditions.

#### ERPs in LAN time window (300–450 ms)

There was an agreement × laterality interaction at fast, *F*_(2, 48)_ = 3.80, *p* = 0.033 (*p* = 0.029), and midfast rates of word presentation, *F*_(2, 48)_ = 4.75, *p* = 0.018 (*p* = 0.015), and a marginal agreement × antpost interaction at slow rate of presentation, *F*_(1, 24)_ = 3.75, *p* = 0.065.

**Figure 2:**
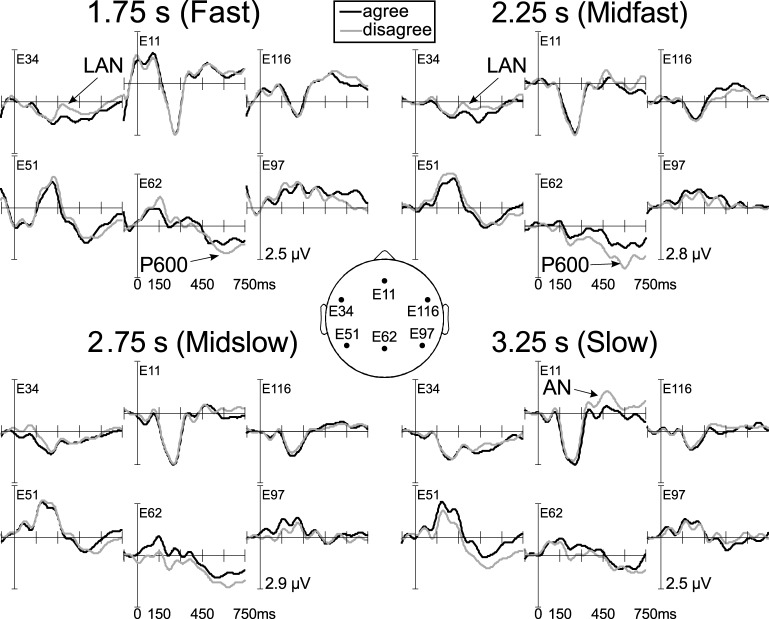
**ERPs from six channels at the four different rates of word presentation for sentence-final adjectives that agree (black line) or disagree (gray line) with the sentence-initial pronoun as regards number.** Between 300 and 450 ms, there was a left-lateralized negativity at fast and midfast rates and a slightly right-skewed anterior negativity at slow rate of word presentation. Disagreement also produced a late posterior positivity at fast and midfast rates (P600). Most closely corresponding 10–20-system channels are: F7, FZ, F8, T5, PZ, and T6.

**Figure 3:**
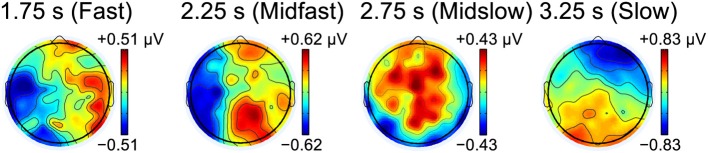
**Topographical distribution of the negativity at the different temporal distances created by the variation in presentation rate, indicated within parentheses.** Average ERPs in the LAN time window (300–450 ms) for the subtraction disagreement–agreement are shown.

At fast, *F*_(1, 24)_ = 6.09, *p* = 0.021, and midfast rates of word presentation, *F*_(1, 24)_ = 6.39, *p* = 0.018, disagreement produced a negativity at left channels. At slow speed of presentation, disagreement yielded a negativity at anterior electrodes, *F*_(1, 24)_ = 4.81, *p* = 0.038 (Figures 2, 3).

**Figure 4:**
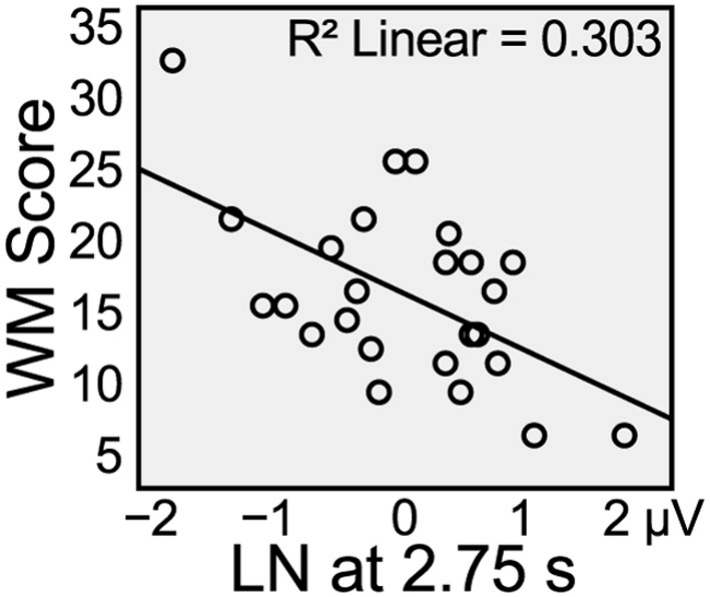
**Correlation between individual working memory span score and average difference between disagreement and agreement ERPs (left negativity, LN) at midslow left channels, 300–450 ms**.

Midslow rate of word presentation did not present any significant effect (Figure 2). However, there was a significant negative correlation between individual working memory span and the average difference between disagreement and agreement for ERPs at left channels, *r* = −0.550, *p* < 0.004 (Figure 4). For comparison, we tested left, mid and right electrode areas at all rates of word presentation, but midslow speed showed the only significant correlation (Bonferroni-corrected).

#### Subdivision of the LAN time window

**Figure 5:**
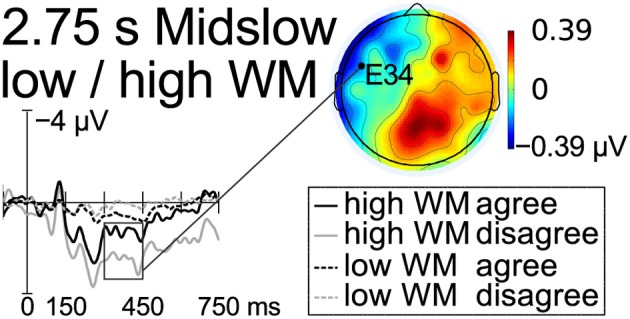
**Results at midslow word presentation rate for two groups scoring highest and lowest on the working memory span test, respectively, at a left anterior electrode.** The negativity at left channels for the high span group in the LAN time window is shown.

To test possible variation within the LAN time window between presentation speeds, we further divided it into three consecutive 50 ms time windows. Fast and midslow presentation rates showed significant effects for left electrodes in the 300–350 ms time window, *F*_(1, 24)_ = 8.02/6.19, *p* = 0.009/0.021, and midslow was also significant in the 400–450 ms time window, *F*_(1, 24)_ = 5.32, *p* = 0.030. At slow rate, the anterior effect was marginal between 300 and 350, *F*_(1, 24)_ = 3.60, *p* = 0.070, and significant only in the 400–450 ms time window, *F*_(1, 24)_ = 9.04, *p* = 0.006. Dividing the participants into groups with the 7 scoring highest and 7 scoring lowest on the working-memory span test showed a significant effect at midslow left electrodes only between 400 and 450 ms, *F*_(1, 6)_ = 6.61, *p* = 0.042 (Figure 5).

### ERPs in late time window (600–750 ms)

There was a marginal agreement × antpost interaction at fast, *F*_(1,24)_ = 3.42, *p* = 0.077, rate of word presentation and an agreement × antpost × laterality interaction at midfast rate of word presentation, *F*_(2, 48)_ = 4.18, *p* = 0.023 (*p* = 0.020).

At fast rate of word presentation, disagreement yielded a positivity at posterior electrodes, *F*_(1, 24)_ = 4.83, *p* = 0.038. At midfast presentation rate, there was a positivity for disagreement at the mid posterior channel area, *F*_(1, 24)_ = 4.60, *p* = 0.042 (Figure 2).

There was no significant effect for midslow or slow presentation rate in this time window.

## Discussion

We tested violation of grammatical number agreement at four different presentation rates, producing different temporal distances between (dis)agreeing words. In the LAN time window (300–450 ms), there was a left-lateralized negativity at the adjective at fast and midfast rates of word presentation, where the distance between the disagreeing pronoun NP and adjective was under 2.5 s. At a time distance of over 3 s (slow presentation rate), the effect was a slightly right-skewed, anterior negativity in this time window. At the midslow rate of word presentation, which resulted in a distance of 2.75 s between disagreeing NP and adjective, there was a negative correlation with individual working memory span, showing a left-lateralized negativity only in participants with greater working memory span.

The results are in accordance with Sachs' (1967, 1974) model of language processing, where the grammatical form of words decays in memory over the first few seconds after exposure, whereas the propositional content of the sentence is maintained. Thus, the presence or absence of a plural suffix on the adjective, e.g., *snäll(-a)* “kind(-pl),” would be thought to trigger participants to search their short-term memory for a matching grammatical form. At a distance up to slightly over 2 s between agreeing words, matching or mismatching grammatical forms were easily found in short-term memory, resulting in detection of morphosyntactic violation indexed by a LAN. When the agreement domain approached 3 s, participants with high working memory spans could be thought to still have the grammatical form present in short-term memory, and therefore a correlation was found between left-lateralized negativity and individual working memory span. However, when the distance between the incongruous words exceeded 3 s, the processing of agreement on the basis of formal features was aggravated and the topographical distribution of the negativity changed, suggesting agreement resolution on the basis of pragmatic inference.

An alternative interpretation of the results would be in terms of a sequence detector (Pulvermüller and Shtyrov, 2003). However, it is uncertain whether the predictive nature of a sequence detector would hold for the case of pronoun-predicate adjective agreement. When processing the pronoun at the beginning of the sentence, the reader cannot predict that an adjective will follow later, since adjectives are not obligatory constituents of sentences. One might, however, consider a “reverse” sequence detector, triggered by the adjective suffix, i.e., the second item in the agreement relation, and enforcing activation of the memory trace of the pronoun if still present.

The right-skewed anterior negativity associated with agreement domains over 3 s long was clearly distinct from the left-lateralized negativity found for agreement domains below 2.5 s long. This could be due to the failure to find a grammatical form of the right word class in short-term memory if grammatical forms fade away during the first seconds after exposure. The right-lateralized anterior negativity might reflect extended search for the pronoun based on the propositional content of the sentence. Indeed, the frontal distribution and tendency toward a right-lateralization could be due to retrieval involving frontal cortex to a larger extent and the use of pragmatic inference, engaging the right hemisphere (St George et al., 1999). Further, a timeline analysis of the LAN time window indicated that the frontal effect for slow speed of presentation had a later onset than the LAN found at faster speeds. Whereas the LAN was significant in the 300–350 ms time window, the right-skewed anterior negativity was not significant until 400–450 ms. This might indicate a slower process involving extended search. A right-lateralized anterior negativity might have been expected for participants scoring low on the working-memory span test, but no effect was found for this group. This might be due to individual variation within this group.

There was a late positivity at the adjective for the two shortest time intervals, at fast and midfast presentation rates, but no significant positivity for the longer time intervals. This positivity is likely to be a P600 effect showing repair of grammatical agreement violation at the short intervals between agreeing words (Friederici, 2002). At the longer time intervals, no repair would seem to take place. This might be an indication that grammatical agreement violations are only repaired when they occur within the same perceptual unit.

The LAN obtained at the adjective in the faster presentation rates in the present study did not show an anterior focus, as has been commonly seen in previous studies on agreement relations. The reason might be that the task did not focus on making judgments on the agreement as such, and therefore retrieval of the preceding pronoun might have been rather non-attentive, thus taxing frontal cortex to a lesser extent. Accordingly, a previous study finding a similar distribution used randomly distributed comprehension questions rather than grammaticality judgments (Coulson et al., 1998). On the other hand, most studies finding a LAN with an anterior focus have used obligatory subject-verb agreement which involves a higher degree of predictability than in subject-predicative adjective agreement, where the adjective is not an obligatory constituent. Increased use of predictive strategies might involve frontal cortex to a higher degree. Indeed, a previous study on agreement in Spanish also found less pronounced anteriority of the LAN for adjectives (Barber and Carreiras, 2005). This study also gives support for the interpretation of the LAN as an effect specifically reflecting the processing of formal grammatical features. When adjectives were presented in isolated word pairs (el/^*^los piano “the-sg/^*^the-pl piano”), a centrally distributed negativity, interpreted as an N400 effect, was observed rather than a LAN. Thus, in the absence of a sentence context, it could be thought that agreement matching was based more on lexical features than on grammatical.

## Conclusions

By varying visual presentation rate of sentence stimuli, grammatical agreement processing produced distinct neurophysiological responses at different temporal distances between agreeing words. Violation of agreement produced a left-lateralized negativity and a P600 at distances less than 2.5 s between (dis)agreeing words, and a right-skewed, anterior negativity at a temporal distance of over 3 s between the same words. The variation in neural response indicates that agreement is processed differently depending on whether the words involved in the agreement relation occur within the same perceptual time unit of 2–3 s (cf. Pöppel, 1997) or not. The results add to previous findings of a rather short time window of 2–3 s for integration of different kinds of formal (grammatical and phonological) information during sentence processing (Sachs, 1967, 1974; Vollrath et al., 1992; Roll et al., 2012).

### Conflict of interest statement

The authors declare that the research was conducted in the absence of any commercial or financial relationships that could be construed as a potential conflict of interest.

## Footnotes

^1^The asterisk (^*^) indicates grammatical violation.

^2^Second person *ni* “you-pl” was excluded since it is sometimes used as an older polite form to refer to singular referents, in which case it agrees with singular adjectives. Third person *de/dom* ‘they’ was excluded since variation in spelling conventions might cause confusion. The pronoun is pronounced *dom* “they,” but is written as *de* in formal texts. If written as *de*, however, some readers are likely to pronounce the word as written rather than as it is pronounced in everyday speech. If using the *dom* spelling, on the other hand, the pronoun might be confounded with its homograph, meaning “judgment.”
